# Molecular Mechanisms of Pulmonary Vascular Remodeling in Pulmonary Arterial Hypertension

**DOI:** 10.3390/ijms17050761

**Published:** 2016-05-18

**Authors:** Jane A. Leopold, Bradley A. Maron

**Affiliations:** 1 Division of Cardiovascular Medicine, Brigham and Women’s Hospital, Harvard Medical School, Boston, MA 02115, USA; bmaron@partners.org; 2 Division of Cardiology, Veterans Affairs Boston Healthcare System, Boston, MA 02132, USA

**Keywords:** pulmonary arterial hypertension, DNA damage, microRNA, metabolism, mitochondria, endothelial-to-mesenchymal transition

## Abstract

Pulmonary arterial hypertension (PAH) is a devastating disease that is precipitated by hypertrophic pulmonary vascular remodeling of distal arterioles to increase pulmonary artery pressure and pulmonary vascular resistance in the absence of left heart, lung parenchymal, or thromboembolic disease. Despite available medical therapy, pulmonary artery remodeling and its attendant hemodynamic consequences result in right ventricular dysfunction, failure, and early death. To limit morbidity and mortality, attention has focused on identifying the cellular and molecular mechanisms underlying aberrant pulmonary artery remodeling to identify pathways for intervention. While there is a well-recognized heritable genetic component to PAH, there is also evidence of other genetic perturbations, including pulmonary vascular cell DNA damage, activation of the DNA damage response, and variations in microRNA expression. These findings likely contribute, in part, to dysregulation of proliferation and apoptosis signaling pathways akin to what is observed in cancer; changes in cellular metabolism, metabolic flux, and mitochondrial function; and endothelial-to-mesenchymal transition as key signaling pathways that promote pulmonary vascular remodeling. This review will highlight recent advances in the field with an emphasis on the aforementioned molecular mechanisms as contributors to the pulmonary vascular disease pathophenotype.

## 1. Introduction

Pulmonary hypertension, defined as a mean pulmonary artery pressure ≥25 mmHg, may be a primary disorder or occur secondary to cardiopulmonary disease or a consequence of other clinical disorders [1]. The World Health Organization recognizes five categories of pulmonary hypertension on the basis of underlying etiology, pathology, and hemodynamic profile (Table 1). Pulmonary arterial hypertension (PAH), also known as World Health Organization Group I pulmonary hypertension, is an insidious disease that is associated with a poor long-term prognosis [1]. PAH is defined hemodynamically by a mean pulmonary artery pressure ≥25 mmHg, a pulmonary artery wedge pressure of <15 mmHg and a pulmonary vascular resistance of >3.0 Wood units [1]. An epidemiological study conducted in the United Kingdom and Ireland have determined that the incidence of PAH is approximately 1.1 per million per year with an estimated prevalence of 6.6 cases per million [2]. Recent survival estimates from the United States Registry to Evaluate Early and Long-Term PAH Disease Management report that incident survival at one and three years is 85% and 63%, respectively [3]. The pathological findings that characterize PAH include hypertrophic distal pulmonary artery remodeling; inflammation, fibrosis, and thrombosis; and neovascularization. In some vessels, these processes lead to obstruction of the vessel lumen and create complex vascular lesions that are known as plexiform lesions and are pathognomonic for PAH [4]. This plexigenic arteriopathy increases pulmonary vascular resistance and pulmonary artery pressure to impose a hemodynamic load on the right ventricle. The right ventricle is undergoes (mal)adaptive remodeling to compensate for the increased hemodynamic stress but is prone to failure leading to premature death. Owing to the attendant morbidity and mortality associated with PAH, attention is increasingly focused on discovery of the cellular and molecular mechanisms that initiate pulmonary artery remodeling to identify new pathways for pharmacotherapeutic intervention. In the current review, we will summarize some of the recent advances in the field with a focus on genetic and epigenetic phenomenon, metabolism and mitochondrial function, mineral and essential element handling, and endothelial-to-mesenchymal transition (EndoMT) as key regulatory pathways involved in pulmonary vascular remodeling and PAH.

## 2. Genetic and Epigenetic Regulation of Pulmonary Arterial Hypertension (PAH)

It has long been recognized that there is a genetic component to PAH with the disease clustering in some families. Patients with this form of the disease are referred to as having heritable PAH while individuals with no determined genetic basis for the disease are referred to as having idiopathic PAH. Heritable PAH, defined as occurring in two or more family members, is an autosomal dominant disorder with severe and rapid progression of the disease phenotype [4]. Mutations in the bone morphogenetic protein receptor 2 (*BMPR2*), a member of the transforming growth factor-β (TGFβ) superfamily, are the most common cause for hereditary PAH and account for ~75% of cases and have also been identified in ~25% of patients with idiopathic PAH [5,6,7]. Mutations in other gene members of the TGFβ superfamily have been identified although they are believed to account for only 1%–3% of cases of PAH. Mutations have been identified in the activin A receptor type II-like 1 (*ACVRL1*), endoglin (*ENG*), and members of the Smad family, including *SMAD1*, *SMAD4*, and *SMAD9* [8].

Whole exome sequencing found rare genetic variants associated with PAH [8]. Using this methodology, variants in the genes for caveolin1 (*CAV1*), which regulates SMAD 2/3 phosphorylation, the potassium channel, subfamily K, member 3 (*KCNK3*), which is expressed by pulmonary artery smooth muscle cells and related to proliferation, and the eukaryotic translation initiation factor 2 alpha kinase 4 (*EIF2AK4*), which has been implicated in pulmonary vaso-occlusive disease in an autosomal recessive manner [9,10,11]. It has also been suggested that there are PAH disease modifier genes owing to the observed sex bias with a predilection for women, incomplete penetrance, and variability in the time to onset of disease. Genome wide association studies located two single nucleotide polymorphisms downstream of cerebellin 2 (*CBLN2*) that were associated with a two-fold increased risk of PAH [12]. Mutations in *KCNA5*, the potassium channel voltage gated shaker-related subfamily A, member 5, which is involved in maintaining the pulmonary artery smooth muscle cell contractile state, have also been identified as a “second-hit” in patients with *BMPR2* mutations [13]. When present, this mutation enhances the effects of the *BMPR2* mutation to cause early onset and severe PAH [8]. While these mutations and variants have been linked to PAH by affecting pathways relevant for pulmonary vascular homeostasis, other genetic and epigenetic mechanisms such as the presence of DNA damage, activation of the DNA damage response, and microRNAs (miR) also influence gene expression and downstream signaling pathways.

## 3. DNA Damage in PAH and the DNA Damage Response

There is evidence of DNA damage and somatic genetic abnormalities in pulmonary vascular cells isolated from patients with PAH. This was demonstrated initially in endothelial cells from plexiform lesions that were shown to have microsatellite instability, a condition of genetic hypermutability [14,15,16]. PAH endothelial cells also exhibit large-scale cytogenetic abnormalities [17]. Examination of DNA isolated from explanted PAH lungs as compared to explanted disease control and non-disease control lungs found mosiac chromosomal abnormalities in PAH lungs. One PAH patient had a chromosomal deletion of *BMPR2*, a known genetic cause of PAH, and somatic loss of chromosome 13, which contains *SMAD8* and, therefore, is a “second hit”. Two female PAH patients were also found to have deletion of the active X chromosome although the relevant genetic factors and signaling pathways affected by this deletion that predispose to PAH are not known. Taken together, these findings suggest that DNA damage in PAH lungs appears to occur at a higher than expected rate [18,19].

It has been suggested recently that DNA damage predates the onset of clinical PAH and is likely an intrinsic property of cells in individuals that are susceptible to the disease [20]. To examine this hypothesis, investigators examined measures of baseline DNA damage in pulmonary artery endothelial cells and circulating peripheral blood mononuclear cells. They found copy number changes in 30.2% of pulmonary artery endothelial cells isolated from explant lungs as compared to only 5.3% in cells isolated from donor lungs. This finding did not correlate with the patient’s disease severity. The pulmonary artery endothelial cells with evidence of chromosomal abnormalities and circulating peripheral blood mononuclear cells also had more DNA damage assessed by measuring chromosome breakage and loss. DNA damage in the endothelial cells also correlated with reactive oxygen species production by the endothelial cells. Interestingly, unaffected relatives of PAH patients had similar evidence of DNA damage in their circulating peripheral blood mononuclear cells indicating that the DNA damage observed in PAH patients was not the result of PAH-specific medications [20].

The DNA damage response is activated in pulmonary arteries isolated from patients with PAH and pulmonary artery smooth muscle cells show evidence of DNA damage (*i.e.*, increased expression of the damage markers 53BP1 and γ-H2AX). There is concomitant activation of poly (ADP ribose) polymerase-1 (PARP-1), which is part of the DNA damage response and contributes to DNA repair by binding to strand breaks in DNA and generating poly(ADP-ribose) at the break site. Activation of PARP-1 increases cell survival and proliferation through a mechanism that involves miR-204-mediated activation of nuclear factor of activated T cells (NFAT) c2 and hypoxia inducible factor-1α (HIF-1α). In a rodent model of pulmonary hypertension, treatment with the PARP inhibitor ABT-888 decreases pulmonary hypertension and limits pulmonary artery hypertrophy despite slightly increasing markers of DNA damage in lung homogenates [18]. The presence of DNA damage in PAH is also associated with rapid downregulation of BMPR2, which, in turn, affects the DNA damage response by regulating expression of the DNA repair gene breast cancer 1 (*BRCA1*). Downregulation of BMPR2, a hallmark of PAH, is therefore associated with a decrease in the DNA damage response that further compromises the genomic integrity of the cells [21].

Next generation sequencing has also identified mutations that are associated with a deficient DNA damage response. Whole exome sequencing in 12 unrelated patients with idiopathic PAH identified rare variants in the topoisomerase DNA binding II binding protein 1 (*TOPBP1*). The protein encoded by this gene binds to double- and single-stranded DNA and inhibits E2F transcription factor 1-mediated apoptosis leading to increased cell survival. Pulmonary artery endothelial cells isolated from idiopathic PAH patient lungs had decreased levels of TopBP1 mRNA and protein compared to controls. Decreased expression of TopBP1 was associated with increased susceptibility to hydroxyurea induced DNA damage and apoptosis with evidence of increased phosphorylated histone-2AX, a marker of DNA strand breaks [19].

## 4. MicroRNAs Regulate Gene Expression in PAH

Owing to their significant role in modulating gene expression, it is not surprising that miRs have been shown to contribute to the pathogenesis of PAH. MiRs are a group of small noncoding RNAs of ~22 nucleotides that silence gene expression by binding to the 3′-untranslated regions of mRNAs and inhibiting translation or promoting degradation of the mRNAs [22]. Several studies performed global surveys of miR expression in order to determine the profile of differentially expressed miRs in PAH. One early study that examined a total of 337 miRNAs, identified six that were upregulated, and only one that was downregulated (miR-204) in explanted PAH lungs as compared to control donor lungs [23]. Another study done via microarray using plasma from eight patients with PAH compared to eight healthy subjects. This study identified differences in 58 miRs between the groups with miR-150 highlighted as the most significantly decreased miR in PAH patient plasma [24]. Examination of miR expression in remodeled pulmonary arteries from patients with severe PAH identified miR-126 and miR-21 levels as being upregulated in plexiform compared to concentric lesions while expression of miR-143/145 and miR-204 were higher in concentric lesions [25]. The role of several miRs, including miR-17-92, miR-143/145, miR-204, miR-214, miR-21, and miR-130/301 in regulating PAH relevant signaling pathways and the disease phenotype has been confirmed in cellular and experimental models of the disease (reviewed in [26,27,28,29,30,31,32,33,34,35,36,37,38,39,40]) (Table 2).

The miR-17-92 cluster has been implicated in vascular remodeling in PAH. In pulmonary artery endothelial cells, exposure to the pro-hypertensive cytokine interleukin-6, which is elevated in patients with PAH, increases expression of the signal transducer and activator of transcription 3 (STAT3) and the miR-17-92 cluster. miR-17-92 was shown to target BMPR2 directly resulting in cell proliferation and apoptosis resistance [31]. The expression pattern of miR-17-92 in pulmonary artery smooth muscle cells in PAH is more complex. In the initial stages of disease, miR-17-92 is transiently upregulated in hypoxia models and promotes cell proliferation. Forced downregulation of miR-17 in the hypoxia mouse model led to attenuation of pulmonary vascular remodeling by inducing p21 to limit proliferation [32]. In later stages of disease, miR-17-92 is downregulated and this is associated with decreased expression of the smooth muscle cell markers smooth muscle 22α, α-smooth muscle actin, and calponin suggesting that miR-17-92 expression is necessary to maintain a differentiated phenotype [26].

The miR 143/145 cluster regulates smooth muscle cell differentiation and is necessary to maintain a contractile phenotype [33,34]. miR-145 mediated inhibition of the transcription factor kruppel-like factor 5 (*KLF5*) leads to upregulation of myocardin and the smooth muscle contractile markers smooth muscle myosin heavy chain, α-smooth muscle actin, and calponin. miR-143, which inhibits E26 transformation-specific domain containing protein Elk-1, also maintains the differentiated phenotype. It is known that miR-143/145 is upregulated by hypoxia and increased levels of miR-143/145 have been demonstrated in pulmonary arteries in experimental and human PAH [34,35]. *In vivo*, manipulation of miR-145 expression does not affect pulmonary vascular contractile function but anti-miR-145 appears to inhibit hypoxia-induced pulmonary hypertension in a mouse model [36].

miR-214 is another miR that is upregulated in the lungs and right ventricle (RV) in multiple preclinical models of PAH but, importantly, appears to have sex-related differences in how it modulates the PAH disease phenotype [27,30]. The miR-214 group of miRs includes four distinct mature miRNAs (miR-199-5p, miR-199-3p, miR-214-5p and miR-214-3p) that originate from a bicistronic transcript. miR-214 is induced by TGF-β in pulmonary artery smooth muscle cells and pri-miR-199/214 is increased in the lung and right ventricle in Sugen5416/hypoxia-exposed mice. Further examination revealed that the miR-199/214 axis was upregulated in the lung and right ventricle of male mice but not in female mice suggesting a sex-specific role of this miR. Additional studies performed in miR-214 whole body knockout mice showed that male mice had a significant increase in right ventricular hypertrophy compared to wild-type mice, an effect that was not seen in the female mice. In this study, target gene analysis found that the miR-214 target phosphatase and tensin homolog (PTEN) was upregulated in the right ventricle in knockout mice [27,30].

In patients with PAH, circulating levels of miR-204 correlate inversely with disease severity indicating that this miR may serve as a candidate biomarker [37]. miR-204 is downregulated by increased levels of angiotensin II and endothelin-1, which are present in patients with PAH. A decrease in miR-204 expression leads to upregulation and activation of STAT3 to promote pulmonary artery smooth muscle cell proliferation and apoptosis resistance [23].

The role of miR-21, which is induced by hypoxia, as a significant disease modifying miR in PAH was shown using a systems biology approach and network analysis. Upregulation of miR-21 was confirmed in several rodent models of pulmonary hypertension and in pulmonary arteries from PAH patients. *In vitro*, hypoxia and BMPR2 signaling increased miR-21 expression in pulmonary artery endothelial cells to regulate RhoB expression as well as Rho-kinase activity. Using a miR-21 knockout mouse, loss of miR-21 was shown to increase pulmonary pressures and augment the pulmonary hypertensive phenotype [38]. Network analysis also identified the miR-130/301 family as an important regulator of the pulmonary hypertension network and further analysis determined that it functioned as a master regulator of cell proliferation signaling pathways regulated by subordinate miR pathways. The miR-130/301 family targeted peroxisome proliferator-activated receptor-γ, which regulated apelin-miR-424/503-fibroblast growth factor 2 signaling in pulmonary artery endothelial cells while in pulmonary artery smooth muscle cells, miR-130/301 regulated STAT3-miR-204 signaling. The coordinated signaling pathways regulated by miR-130/301 promote endothelial dysfunction and smooth muscle cell proliferation. This was confirmed in mouse models where induction of miR-130/301 promoted acquisition of a pulmonary hypertension phenotype while inhibition of miR-130/301 prevented pulmonary hypertension [39,40].

Although the aforementioned miRs have been examined in some depth in PAH, it is important to note that miRs have different expression patterns among the cell types important for PAH. Each miR also targets a large number of mRNAs, the miRs may be involved in feedback loops to regulate mRNA expression, and there are likely species-related differences. Thus, failure to identify a specific miR in an experimental model does not exonerate it from playing a role in human disease. Furthermore, when miRs or antagomiRs are used as therapeutics, the off-target effects of manipulating miR expression have not been well studied.

## 5. Changes in Cellular Metabolism, Metabolic Flux, and Mitochondrial Function

**Figure 1:**
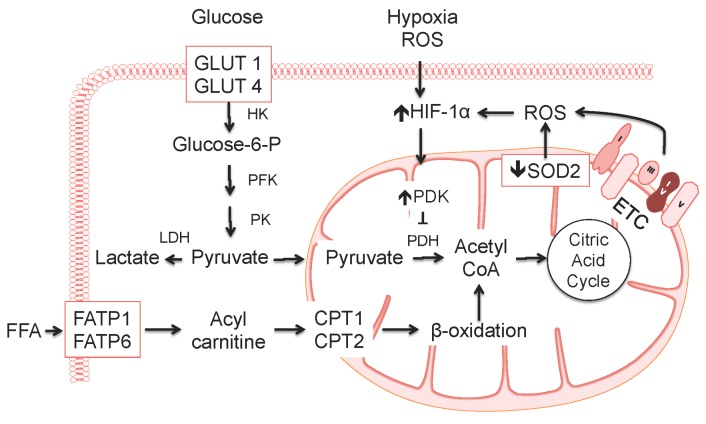
Metabolism in PAH. Metabolism in PAH is perturbed akin to what is observed in cancer. Glycolysis occurs when glucose is taken up by the glucose transporters-1 (GLUT-1) and -4 (GLUT-4), gets phosphorylated by hexokinase (HK), and goes through a series of reactions to produce pyruvate. Pyruvate is the substrate for pyruvate dehydrogenase (PDH) in the mitochondria to support glucose oxidation. Free fatty acids (FFA) are taken up by fatty acid transport protein-1 (FATP-1) and -6 (FATP-6) and transformed to acyl carnitines that are shuttled across the mitochondrial membrane by carnitine palmitoyltransferase-1 (CPT1) and transformed to acyl CoA by carnitine palmitoyltransferase-2 (CPT2). Acyl CoA is converted to acetyl CoA during β-oxidation. In PAH, there is increased aerobic glycolysis due to normoxic upregulation of HIF-1α, which upregulates pyruvate dehydrogenase kinase (PDK) to inhibit pyruvate dehydrogenase, and epigenetic regulation of the superoxide dismutase 2 (*SOD2*) gene. PFK, phosphofructokinase; PK, pyruvate kinase; LDH, lactate dehydrogenase; ROS, reactive oxygen species; ETC, electron transport chain.

Cellular metabolism is a dynamic process that can change rapidly to respond to the (patho)physiological environment. Glycolysis occurs in the cell cytosol and generates pyruvate that is transported to the mitochondria where it serves a substrate for pyruvate dehydrogenase as part of glucose oxidation (Figure 1). When pyruvate dehydrogenase is inhibited, there can be uncoupling of glycolysis from glucose oxidation leading to an increase in intracellular lactate levels (reviewed in [41]). The mitochondria are also the site of fatty acid β-oxidation to yield ATP and acetyl coenzyme A for entry into the citric acid cycle [42]. There is metabolic crosstalk between glycolysis and fatty acid oxidation such that a change in the activity of one pathway influences the activity of the other. This is exemplified by the Randle cycle where a reciprocal relationship was discovered between glucose oxidation and fatty acid oxidation [43]. The Warburg effect, or aerobic glycolysis, is a shift in metabolism to glycolysis followed by fermentation of lactic acid instead of pyruvate oxidation in the mitochondria. The net gain from this metabolic shift, which was first described in cancer cells, is to produce ATP to meet energy requirements and to promote cell growth and survival [44]. The shift to aerobic glycolysis usually occurs when a key mitochondrial metabolic enzyme such as pyruvate dehydrogenase or pyruvate dehydrogenase kinase is inhibited. Mitochondrial function is also reliant upon its structural integrity. Within cells, mitochondria continuously divide and join together in processes referred to as fission and fusion. The mitochondria are also important for oxygen sensing and this is dependent, in part, on the stability of the mitochondrial network (reviewed in [45]).

Metabolism is perturbed in PAH with consequences for cell proliferation, survival, and apoptosis. In PAH, pulmonary vascular cells demonstrate increased aerobic glycolysis as a result of normoxic upregulation of HIF-1α and inhibition of pyruvate dehydrogenase. The pseudohypoxic state that leads to normoxic activation of HIF-1α occurs due to changes in redox state [46,47,48]. The cellular redox state is affected by decreases in the levels of mitochondrial reactive oxygen species generation that inhibits oxidative metabolism [49]. Mitochondria in PAH are fragmented due to activation of the fission regulator dynamin-related protein 1, have decreased expression of both complex I and superoxide dismutase, and have a hyperpolarized membrane [49]. Mitochondrial superoxide dismutase can by repressed transcriptionally via methylation of 2 CpG islands in the *SOD2* gene. Interestingly, this phenomenon occurs in the lung vessels only and is not observed in the systemic circulation. This finding is likely due to higher levels of DNA methyltransferases in the lung [50]. Normoxic activation of HIF-1α in PAH upregulates pyruvate dehydrogenase kinase isoforms 1 and 2 leading to phosphorylation and inhibition of pyruvate dehydrogenase with a switch to aerobic glycolysis. The small molecule dichloroacetate, which is a pyruvate dehydrogenase kinase inhibitor, has shown promise as a potential therapy in experimental pulmonary hypertension. Dichloroacetate improves mitochondrial structural integrity and function, decreases pulmonary artery smooth muscle cell proliferation, and regresses established pulmonary hypertension [51,52,53,54].

## 6. Zinc, Iron, and Calcium Handling in Pulmonary Hypertension

In addition to shifts in metabolic pathways in PAH, there is also evidence of alterations in zinc, iron, and calcium handling in the disease. These minerals and ions are essential components of proteins, function as cofactors, and are involved in pulmonary artery cellular homeostasis. It has recently been shown that zinc is an important contributor to pulmonary artery smooth muscle cell proliferation, distal pulmonary artery remodeling, and pulmonary hypertension. Using linkage analysis and whole genome sequencing, investigators were able to identify *Slc39a12*, which encodes solute carrier 39 zinc transporter family member 12 (ZIP12), as a candidate susceptibility gene for hypoxic pulmonary hypertension. ZIP12 is a member of the zinc transporter protein (ZIP) family, which regulates cellular zinc levels by transporting zinc from the extracellular to the intracellular compartment. In rats exposed to hypoxia that develop pulmonary hypertension, ZIP12 mRNA and protein expression were upregulated in the lungs compared to normoxic controls. This finding was also observed in cattle with naturally occurring pulmonary hypertension, or “brisket disease”, and humans at high altitude exposed to chronic hypoxia. *In vitro*, pulmonary artery smooth muscle cells exposed to hypoxia expressed increased levels of ZIP12, elevated levels of intracellular labile zinc, and increased proliferation. When ZIP12 was inhibited, hypoxia-stimulated smooth muscle cell proliferation was abrogated. Genetic mutation of ZIP12 in rats exposed to hypoxia (10%) for two weeks was associated with lower pulmonary artery pressures and less pulmonary artery remodeling than control rats [55].

Clinical studies have found that iron deficiency (but no anemia) in PAH patients is linked to decreased exercise capacity and survival. To explore this relationship, rats were fed an iron-deficient diet for a month and decreases in serum iron, iron levels in the liver, and ferritin levels were confirmed. These iron-deficient animals developed pulmonary hypertension with hypertrophic pulmonary vascular remodeling and inflammation. Signaling intermediaries relevant for smooth muscle cell proliferation, including HIF-1α, STAT3, and NFAT, were upregulated in the remodeled pulmonary vessels. Mitochondria isolated from rats on the low iron diet were functionally abnormal and demonstrated decreased complex I activity and mitochondrial membrane hyperpolarization. Pulmonary hypertension was reversed by iron replacement therapy suggesting that iron is a key mineral that is required to maintain pulmonary vascular homeostasis [56]. Iron replacement therapy has also been trialed in patients with PAH; however, while it improved exercise endurance and aerobic capacity, iron replacement did not significantly change the six-minute walk distance at 12 weeks [57].

Calcium signaling is also important in the pathogenesis of PAH owing to its role in pulmonary artery smooth muscle cell proliferation and vasoconstriction. Increased cytosolic calcium is a major stimulator of smooth muscle cell proliferation by activating Ca^2+^-dependent kinases, immediate early genes, and NFAT [58]. Calcium influx via the plasma membrane and release from intracellular stores are the main regulators of cytosolic calcium levels. In pulmonary artery smooth muscle cells, L- and T-type calcium channels are important for voltage-gated calcium entry and are linked to proliferation [55,59]. Receptor- and store-operated calcium channels also modulate intracellular calcium homeostasis and are important for excitation-contraction coupling. The sarcoplasmic reticulum releases calcium stores to initiate actin-myosin interactions. Once calcium is depleted from the sarcoplasmic reticulum, the sarco/endoplasmic reticulum Ca^2+^-ATPase (SERCA) sequesters calcium into the sarcoplasmic reticulum and replenishes calcium stores [60,61,62].

SERCA2 is downregulated in smooth muscle cells following vascular injury and is associated with dedifferentiation of these cells to a proliferative phenotype. In PAH, SERCA2 expression is decreased in remodeled human pulmonary arteries compared to disease controls. To determine if restoring SERCA2 levels would be therapeutic in pulmonary hypertension, gene transfer of human SERCA2a using an adeno-associated virus serotype 1 (AAV1.SERCA2a) via aerosolized inhalation was performed in the rat monocrotaline model and the porcine pulmonary vein banding model of pulmonary hypertension [63,64,65]. In these studies, increased expression of SERCA2a was detected in the pulmonary arteries and was associated with decreased pulmonary artery pressure, decreased pulmonary artery medial thickness, and improved right ventricular function. Increasing SERCA2a expression in pulmonary artery smooth muscle cells resulted in decreased activation of STAT3 and NFAT signaling as well as a trend towards increased BMPR2 expression. Overall, these studies indicate that restoring intracellular calcium handling is beneficial in experimental pulmonary hypertension and that gene transfer of SERCA2a is an effective therapeutic in the disease [63,64,65].

## 7. Endothelial-to-Mesenchymal Transition

**Figure 2:**
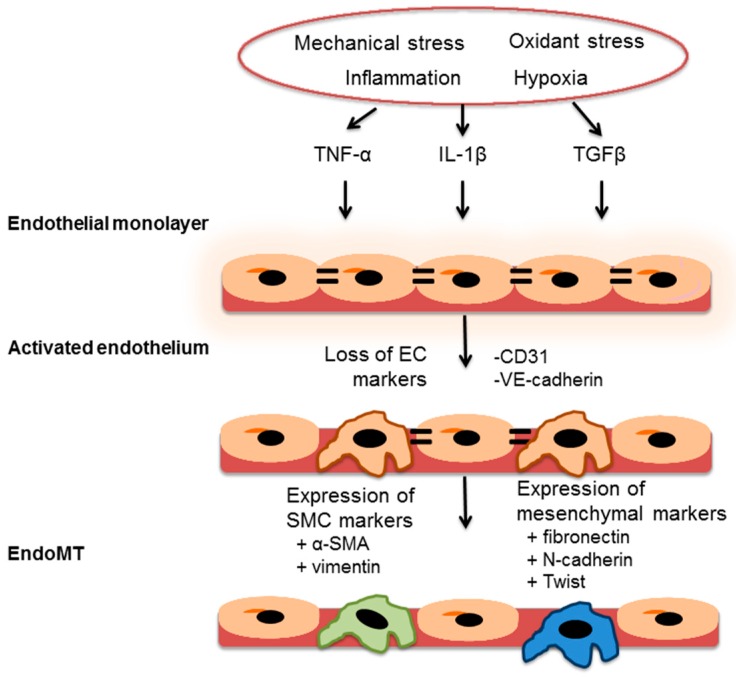
Endothelial-to-mesenchymal transition. Endothelial-to-mesenchymal transition (EndoMT) occurs when the endothelium is exposed to environmental stressors that increase levels of transforming growth factor-β (TGFβ), tumor necrosis factor-α (TNF-α), or interleukin-1β (IL-1β). These factors activate a select population of endothelial cells, which lose their endothelial markers (*i.e.*, CD31, vascular endothelial cadherin (VE-cadherin), and von Willibrand factor (vWF)) and lose tight gap junctions between cells (double lines). These endothelial cells then express α-smooth muscle actin (α-SMA) and vimentin. Cells may also acquire a mesenchymal phenotype and express fibronectin, N-cadherin, and the EndoMT-related transcription factor Twist.

It has been hypothesized that endothelial-to-mesenchymal transition (EndoMT) occurs in PAH and that these transformed endothelial cells are the source of some of the α-smooth muscle actin positive cells that accumulate in the medial wall and participate in hypertrophic remodeling [66]. EndoMT is a process by which endothelial cells exhibit phenotype plasticity and acquire properties of myofibroblast or mesenchymal cells (Figure 2). This process of phenotype transition is associated with loss of tight gap junctions, dissociation from the basement membrane, and migration into the medial layer. During this process, the cells lose their typical endothelial markers, such as CD31 and vascular endothelial cadherin, and start to express α-smooth muscle actin and vimentin [66]. EndoMT is stimulated by members of the TGFβ superfamily that signal through canonical (Smad 2/3) and noncanonical (ERK 1/2 and p38MAPK) pathways. EndoMT may also occur after exposure to inflammatory molecules, such as interleukin-1β and tumor necrosis factor-α, or angiotensin II receptor type-1 activation [67]. In patients with PAH, examination of pulmonary artery plexiform lesions revealed that luminal endothelial cells were swollen and some cells expressed both endothelial markers and α-smooth muscle actin, a finding not observed in controls. The presence of EndoMT was also confirmed in PAH lungs by showing expression of mesenchymal genes, including fibronectin, N-cadherin, and vimentin and the EndoMT-related transcription factor Twist [68].

In the SU-5416/hypoxia model of pulmonary hypertension, investigators found that 6% ± 1% of endothelial cells in pulmonary arteries were positive for α-smooth muscle actin, suggesting EndoMT. This phenomenon was also observed in explanted lungs from patients with systemic sclerosis-associated PAH where EndoMT was present in 4% ± 1% of pulmonary arterioles examined with none observed in control non-PAH lungs. *In vitro*, otherwise healthy pulmonary artery endothelial cells exposed to inflammatory cytokines underwent EndoMT with a loss of typical endothelial markers and expression of α-smooth muscle actin, calponin, and collagen type I. These transformed endothelial cells also acquired a pro-inflammatory phenotype with increased secretion of inflammatory cytokines (interleukins-4, -13, -6, and -8, and tumor necrosis factor-α). These cells failed to form effective barriers due to loss of tight junctions rendering them primed for proliferation and migration and permissive for leukocyte infiltration [69].

Fate mapping of endothelial cells in pulmonary hypertension has also been done. These studies indirectly confirm that EndoMT occurs and contributes to the origin of neointimal cells in human plexiform lesions. Using mTomato/mGreen double-fluorescent reporter mice to examine endothelial genetic lineage investigators found that endothelial lineage marked cells were present at high levels in the neointima of remodeled pulmonary arteries. Many of these endothelial cells were shown to express α-smooth muscle actin and smooth muscle myosin heavy chain consistent with EndoMT [70].

The other source of pulmonary artery smooth muscle cells involved in pathogenic remodeling of the distal pulmonary arteries derives from pre-existing smooth muscle cell marker positive cells. The cells involved in this process undergo dedifferentiation, migration to the distal vessel, proliferation, and then redifferentiation, similar to what is observed in pulmonary artery development [71]. It has also been shown that smooth muscle cell progenitors are involved in the muscularization of distal pulmonary vessels. These progenitors stain express both smooth muscle cell and undifferentiated mesenchymal cell markers. Under hypoxic conditions, kruppel-like factor 4 (KLF4) expression is upregulated and cells are stimulated to migrate distally, dedifferentiate, and then expand clonally giving rise to new distal smooth muscle cells that muscularize the vessel. This pathway was confirmed in patients with pulmonary hypertension where increased KLF4 expression was observed in proliferating distal pulmonary artery smooth muscle cells [72].

## 8. Conclusions

The diverse cellular and molecular pathways that are operative in PAH converge to generate a pathophenotype that is characterized by distal pulmonary artery hypertrophic remodeling that increases pulmonary vascular resistance and pulmonary artery pressure. While heritable causes of PAH continue to be identified with next generation sequencing studies, it is now appreciated that DNA damage, an impaired DNA damage response, and miRs may be equally important epigenetic phenomena that regulate the pulmonary vascular phenotype in PAH. Other studies have demonstrated that changes in pulmonary vascular cell metabolism and mitochondrial function resemble what is observed in cancer cells. This metabolic shift likely contributes to the dysregulated cell proliferation that is observed during pulmonary artery remodeling. This may also underlie the endothelial phenotype transition that occurs with EndoMT where endothelial cells assume a smooth muscle or mesenchymal cell profile and contribute to neointimal formation and muscularization of pulmonary arterioles. Despite the heterogeneity in the signaling pathways related to each of the aforementioned contributors to the pulmonary vascular phenotype in PAH, it is likely they function in concert to promote pulmonary vascular remodeling and pulmonary hypertension. This suggests that future therapeutic interventions should target multiple signaling pathways to ameliorate the aberrant vascular remodeling that occurs in PAH.

## Figures and Tables

**Table 1:** Classification of pulmonary hypertension.

WHO Group	Clinical Group	Clinical Definition	Hemodynamic Definition
1	Pulmonary arterial hypertension	Precapillary PH	mPA ≥ 25 mmHg
			mPAWP < 15 mmHg
2	PH due to left heart disease	Postcapillary PH	mPA ≥ 25 mmHg
			mPAWP > 15 mmHg
		Isolated postcapillary PH	DPG < 7 mmHg and/or
			PVR ≤ 3 Wood units
		Combined postcapillary and precapillary PH	DPG < 7 mmHg and/or
			PVR ≥ 3 Wood units
3	PH due to lung disease or hypoxia	Precapillary PH	mPA ≥ 25 mmHg
			mPAWP < 15 mmHg
4	Chronic thromboembolic pulmonary hypertension	Precapillary PH	mPA ≥ 25 mmHg
			mPCWP < 15 mmHg
5	PH associated with miscellaneous diseases	Precapillary PH	mPA ≥ 25 mmHg
			mPAWP < 15 mmHg
		Postcapillary PH	mPA ≥ 25 mmHg
			mPAWP > 15 mmHg
		Isolated postcapillary PH	DPG < 7 mmHg and/or
			PVR ≤ 3 Wood units
		Combined postcapillary and precapillary PH	DPG < 7 mmHg and/or
			PVR ≥ 3 Wood units

WHO, World Health Organization; PH, pulmonary hypertension; mPA, mean pulmonary artery pressure; mPAWP, mean pulmonary artery wedge pressure; DPG, diastolic pulmonary gradient.

**Table 2:** MicroRNA expression in pulmonary arterial hypertension (PAH).

MicroRNA	Expression in PAH	Species and Model	Reference
miR-17-92	↑	Mouse—hypoxia	[27,32]
		Rat—monocrotaline, hypoxia	
miR-21	↑	Mouse—hypoxia, Sugen5416/hypoxia, *VHL* null	[25,38]
		Interleukin-6 transgenic	
		Rat—monocrotaline	
		Human PAH—pulmonary arteries, plexiform lesions	
miR-126	↓	Rat—monocrotaline	[29]
		Human PAH—right ventricle	
miR-145	↑	Mouse—hypoxia, *BMPR2* mutation	[25,36]
		Human PAH—lung tissue, plexiform lesions	
miR-150	↓	Human PAH—plasma	[24]
miR-204	↓	Mouse—hypoxia	[23,25,37]
		Rat—monocrotaline, Sugen5416/hypoxia	
		Human PAH—lung, pulmonary arteries	
miR-210	↑	Mouse—Sugen5416/hypoxia	[28]
		Human PAH—pulmonary arteries	
miR-214	↑	Mouse—hypoxia, Sugen5416/hypoxia	[27,30]
		Rat—monocrotaline, Sugen5416/hypoxia	
miR-130/310	↑	Mouse—hypoxia, Sugen5416/hypoxia, *VHL* null, Interleukin-6 transgenic, *BMPR2X* transgenic, *Schistosoma mansoni*-infected	[39,40]
		Rat—monocrotaline	
		Juvenile lamb—pulmonary artery-aorta shunt	
		Human PH—pulmonary artery plasma	
